# Microencapsulation of Essential Oils Using Faba Bean Protein and Chia Seed Polysaccharides via Complex Coacervation Method

**DOI:** 10.3390/molecules29092019

**Published:** 2024-04-27

**Authors:** Alicja Napiórkowska, Arkadiusz Szpicer, Elżbieta Górska-Horczyczak, Marcin Andrzej Kurek

**Affiliations:** Department of Technique and Food Development, Warsaw University of Life Sciences, 02-787 Warsaw, Poland; arkadiusz_szpicer@sggw.edu.pl (A.S.);

**Keywords:** essential oil, faba bean, chia mucilage, complex coacervation

## Abstract

The aim of this study was to develop microcapsules containing juniper or black pepper essential oils, using a combination of faba bean protein and chia seed polysaccharides (in ratios of 1:1, 1:2, 2:1). By synergizing these two polymers, our goal was to enhance the efficiency of essential oil microencapsulation, opening up various applications in the food industry. Additionally, we aimed to investigate the influence of different polymer mixing ratios on the properties of the resulting microcapsules and the course of the complex coacervation process. To dissolve the essential oils and limit their evaporation, soybean and rapeseed oils were used. The powders resulting from the freeze-drying of coacervates underwent testing to assess microencapsulation efficiency (65.64–87.85%), density, flowability, water content, solubility, and hygroscopicity. Additionally, FT-IR and DSC analyses were conducted. FT-IR analysis confirmed the interactions between the components of the microcapsules, and these interactions were reflected in their high thermal resistance, especially at a protein-to-polysaccharide ratio of 2:1 (177.2 °C). The water content in the obtained powders was low (3.72–7.65%), but it contributed to their hygroscopicity (40.40–76.98%).

## 1. Introduction

Complex coacervation is a method of creating microcapsules in which a homogeneous colloidal solution of protein and polysaccharide is utilized to form a shell around active substances. This process occurs below the isoelectric point characteristic of the given protein. Under such pH conditions, the protein is positively charged, while the polysaccharides remain negatively charged, leading to molecular associative phase separation into coacervate and supernatant phases [1,2]. Various types of proteins are used in the process of complex coacervation, most commonly gelatin [3,4], milk proteins [5,6], soy protein [7,8], or pea protein [9,10]. However, broad bean protein is less popular. To our knowledge, no attempt has been made to use this protein in complex coacervation so far.

Broad beans (*Vicia faba* var. major), also referred to as vetch broad beans, faba beans, or fava beans, are an annual legume plant belonging to the *Fabaceae* family [11]. The popularity of this plant is on the rise, largely due to its high protein content in the seeds, approximately 32.2% [12], and even higher protein content in isolates, approximately 71.6% [13]. Faba bean protein exhibits good solubility in water and the ability to form stable emulsions [14]. Moreover, a broad bean protein isolate requires the lowest concentration to form a gel when compared to other plant-based proteins [15]. These attributes position it as a promising wall material in microcapsules produced through complex coacervation. Additionally, chia seed (*Salvia hispanica* L., *Lamiaceae* family) polysaccharides have emerged as another key component in complex coacervation. Mucus extracted from chia seeds is a complex anionic heteropolysaccharide resulting from the contact of the seeds with water. Even in low concentrations, this mucus increases the viscosity of the solution and has thickening, emulsifying, or stabilizing properties [16,17]. The combination of broad bean protein and chia seed polysaccharides seems to be promising in the context of microencapsulation of essential oils.

Essential oils are garnering growing attention in the food industry for their antimicrobial properties, which offer the potential for food preservation. However, their application is often restricted by their intense taste and aroma. Being volatile compounds, highly sensitive to environmental factors like light, temperature, and pH, which can induce changes in their chemical composition, essential oils present a challenge related to storage. Consequently, there is a quest for preservation methods, and microencapsulation using complex coacervation emerges as a promising approach [18,19].

The aim of this study was to fabricate microcapsules containing essential oil from juniper or black pepper, utilizing a combination of faba bean protein and chia seed polysaccharides. By synergizing those two polymers, our objective was to enhance the efficiency of microencapsulation of essential oils, thereby paving the way for diverse applications in the food industry. We used soybean and rapeseed oils as core materials to dissolve essential oils to limit their evaporation during the preparation of coacervates and microcapsules. Black pepper and juniper essential oils were selected for their potential health and aromatic properties. Black pepper oil is known for its antibacterial, anti-inflammatory, and stimulating properties. In turn, juniper oil is valued for its disinfecting, anti-inflammatory, and antiseptic properties. Their use in microcapsules may enable easier and more effective use of these beneficial properties in various applications, such as the pharmaceutical, cosmetics, and food industries. Additionally, we aimed to investigate the impact of different polymer mixing ratios on the properties of the resulting microcapsules and the course of the complex coacervation process.

## 2. Results and Discussion

The study examined twelve distinct samples, categorized into four groups based on three factors: the type of oil (soybean or rapeseed), the type of essential oil (juniper or black pepper), and the mixing ratio of broad bean protein with chia polysaccharides (1:1, 1:2, and 2:1). Comparisons were conducted within each of these four groups, facilitating an assessment of how individual components influenced the ultimate characteristics of the microcapsules.

### 2.1. Microcapsule Yield and Encapsulation Efficiency

Across all variants, the microcapsule yield remained consistent, ranging from 48.64% to 49.31% (Table 1). Specifically, the RB1 sample exhibited the lowest MY, while the RB2 sample demonstrated the highest value. Statistical analysis showed that all factors and interactions between them significantly influenced the obtained MY values (*p* ≤ 0.001) (Table 1).

The encapsulation efficiency ranged from 65.64% to 87.85% (Table 1). Notably, the RB2 sample exhibited the highest EE value, coinciding with its superior efficiency in microcapsule production. Across all instances, samples with an FB/CHP ratio of 1:2 consistently demonstrated higher EE compared to those with other mixing ratios. Factorial ANOVA showed that MR and the EO content in the microcapsules had a statistically significant impact on EE (*p* ≤ 0.05) (Table 1).

The mixing ratio of biopolymers plays a crucial role in determining the distribution of electric charges and subsequently influences the balance of charges and electrostatic interactions during the coacervation process [20]. Surprisingly, in this study, the analysis revealed that the mixing ratio did not exert a statistically significant effect on the outcome. This suggests that other factors related to the coacervation process (pH, temperature) itself may have overshadowed the impact of the biopolymer ratio.

### 2.2. Density, Carr Index, and Hausner Ratio

The bulk density of the samples ranged from 0.11 to 0.16 g/cm^3^ (Table 1), with RB3 and SJ1 exhibiting the highest density and RB2 and SJ2 showing the lowest. Statistical analysis revealed that all factors and their interactions had a statistically significant impact on the ρ_bulk_ values obtained (*p* ≤ 0.001) (Table 2). Tapped density was in the range of 0.20 to 0.30 g/cm^3^ (Table 1), approximately twice as high as the initial ρ_bulk_ [21]. All factors significantly influenced the values, albeit to different extents. Specifically, the content of oil, essential oil, and the interactions between oil content and MR, as well as oil content and EO, exhibited a greater impact (*p* ≤ 0.001) compared to the interactions between MR and EO and all factors concurrently (*p* ≤ 0.05) (Table 1). These findings indicate that the complex coacervation process led to a significant reduction in bulk density compared to the individual components. Faba bean protein typically has a ρ_bulk_ of around 0.95 g/cm^3^ [22], while CHP ranges between 0.472 and 0.567 g/cm^3^ [23]. Therefore, the observed decrease in bulk density suggests successful microcapsule formation, with the coacervation process effectively reducing the density of the resulting samples. The decrease in bulk density, while indicative of successful microcapsule formation, may have a negative impact from a storage perspective. Lower density results in increased volume for a given mass, potentially posing challenges in storage and transportation due to the larger space requirements [24]. An increase in tapped density compared to bulk density is a common occurrence, typically resulting in roughly a two-fold increase. This happens due to a more ordered arrangement of material particles during the tapping process, reducing the space between them. In the microcapsules we acquired, this difference was approximately two-fold, likely due to their low water content (3.3%, Table 1) [21]. However, the disparity between tapped and bulk densities can vary, depending on factors like water content, particle size, and the type of wall material. In a study by Airouyuwa and Kaewmanee [25], the bulk and shaken densities of microcapsules that were based on pea protein and contained moringa leaf extract were 0.35 and 0.40 g/cm^3^, respectively. Despite the water content in these microcapsules being similar to that obtained in our tests (ranging from 5.21% to 7.40%), the difference in density values was small. Ultimately, comprehending these differences necessitates considering various process factors and material properties.

The Carr Index ranged from 45.81% to 48.01% (Table 1), while the Hausner ratio spanned from 1.82 to 1.92 (Table 1). In both cases, individual factors and their interactions, as well as the interaction between MR and EO, significantly influenced the CI and HR values (*p* ≤ 0.001). However, the interaction between oil content and MR did not show statistical significance. Conversely, the interaction between oil and EO content was statistically significant only concerning the CI values (*p* ≤ 0.05) (Table 1). The SJ2 sample exhibited the lowest values for CI and HR, while the SB2 sample showed the highest values. According to the interpretation of CI and HR values, all microcapsules obtained exhibited very poor or virtually no flow characteristics [26]. This aligns with the typical attributes of microcapsules produced via complex coacervation and freeze-drying methods [27,28]. Based on the obtained results, it cannot be concluded that any specific mixing ratio is superior in terms of the resulting CI and HR values. Further optimization of the coacervation process may be required to enhance the flow properties of the microcapsules and facilitate their handling and processing in practical applications.

### 2.3. Solubility, Moisture Content, and Hygroscopicity

**Figure 1:**
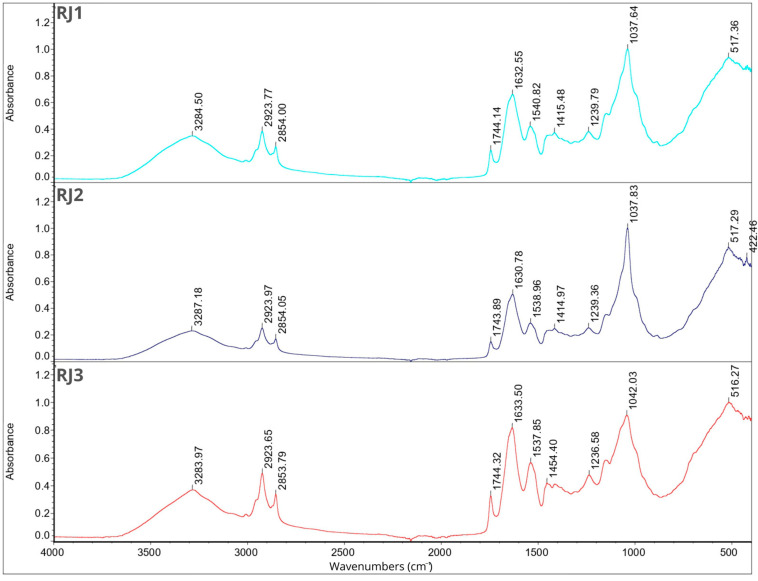
FT-IR spectra for samples RJ1, RJ2, RJ3.

The solubility of the microcapsules in water was found to be low and exhibited a strong dependency on the FB/CHP ratio, ranging from 8.53% to 38.38% (Table 1). Samples with a 1:1 ratio (RJ1, RB1, SJ1, and SB1) displayed the lowest solubility, with values ranging from 8.53% to 13.51%, while those with a 1:2 ratio (RJ2, RB2, SJ2, and SB2) demonstrated higher solubility, ranging from 28.88% to 38.38% (Table 1). Factorial ANOVA showed that the oil content and the interaction between oil and essential oil content had the greatest impact on the solubility of microcapsules (*p* ≤ 0.001). The EO content also influenced the solubility but to a lesser extent (*p* ≤ 0.05). The remaining factors and the interactions between them were not statistically significant (Table 1). The moisture content in the samples was generally low, ranging from 3.72% to 7.65%. However, three samples (RB2, SJ2, and SB3) exhibited higher MC, exceeding 5%, with values of 7.65%, 5.02%, and 7.46%, respectively. There was no statistically significant impact of any of the tested parameters on the MC value (Table 1). Interestingly, the water content showed a correlation with density, with density decreasing as moisture content increased (Figure 1). These findings suggest that the observed changes in bulk and tapped densities with increasing moisture content indicate the presence of strong inter-particle liquid bridges and interlocking forces between particles, as reported in previous studies [21]. The hygroscopicity of the samples ranged from 40.40% to 76.98% (Table 1), with the RB3 sample exhibiting the lowest value and the RB2 sample the highest. This parameter showed a direct correlation with water content, increasing with higher moisture content [29]. Such a relationship is likely due to non-covalent interactions between the microcapsule components and water molecules in the environment, including hydrogen bonds or van der Waals forces [30]. The FT-IR analysis revealed the presence of functional groups (-OH, -C=O, -COOH, -NH_2_) capable of electrostatically interacting with water molecules, which may explain this observed correlation. Nevertheless, factorial ANOVA showed a statistically significant impact (*p* ≤ 0.001) of the oil content in the sample on the H value.

### 2.4. Color Measurement

Based on the findings, it can be inferred that the microcapsules exhibited a hue akin to that of the wall components, indicating that their color primarily relied on the concentration of these constituents within the sample (Table 1). Statistical analysis showed that the L* parameter was statistically significantly influenced by oil content, mixing ratio, EO content, and interactions between oil I EO and MR I EO (*p* ≤ 0.001). The remaining parameters and interactions were not statistically significant. The L* parameter values for the microcapsules spanned from 87.85 to 95.93, signifying their light complexion. Meanwhile, the a* parameter ranged from 0.90 to 1.56, and the b* parameter from 7.50 to 9.91. The parameter a* was significantly influenced by MR and the interaction between oil content and MR (*p* ≤ 0.001). The remaining parameters were not significant (Table 1). In turn, parameter b* was significantly influenced by the content of oil and EO as well as the interaction between the content of oil and MR (*p* ≤ 0.001). The mixing ratio had a significant impact on the b* value, but to a lesser extent (*p* ≤ 0.05) (Table 1). The remaining parameters were not significant. A comparison of these values with the color parameters for broad bean protein (L = 96.67, a = 0.65, b = 6.02 [22]) and chia polysaccharides (L = 87.23, a = 1.76, b = 10.59 [31]) revealed that the microcapsules were lighter than chia polysaccharides (higher L* value) and exhibited a more yellowish tone (higher b* parameter value) than broad bean protein. Hence, it can be inferred that these ingredients influenced the ultimate hue of the microcapsules, with a greater proportion of broad bean protein resulting in a lighter shade and a higher concentration of chia polysaccharides imparting a more yellowish tint. Additionally, it was observed that the presence of RSO, SBO, JEO, or BPO did not exert a statistically significant influence on the color.

**Table 1:** Microcapsule yield (MY), encapsulation efficiency (EE), solubility (S), hygroscopicity (H), moisture content (MC), bulk and tapped densities (ρ_bulk_, ρ_tap_), Carr Index (CI), Hausner ratio (HR), color parameters (L*, a*, b*), and PDI values.

Sample	MY [%]	EE [%]	S [%]	H [%]	MC [%]	ρ_bulk_ [g/cm^3^]	ρ_tap_ [g/cm^3^]	CI [%]	HR	L*	a*	b*	SI
RJ1	49.27 ± 0.00 ^a^	83.23 ± 6.99 ^ab^	12.58 ± 0.10 ^a^	62.74 ± 0.00 ^abcd^	4.78 ± 0.20 ^a^	0.13 ± 0.00 ^a^	0.24 ± 0.01 ^a^	45.83 ± 0.34 ^a^	1.85 ± 0.01 ^a^	90.91 ± 1.93 ^a^	1.53 ± 0.08 ^ef^	8.91 ± 0.54 ^a^	0.47 ± 0.00 ^d^
RJ2	48.89 ± 0.02 ^e^	84.61 ± 6.68 ^abc^	28.88 ± 0.10 ^a^	61.94 ± 0.01 ^bcd^	4.20 ± 0.23 ^a^	0.14 ± 0.00 ^b^	0.24 ± 0.00 ^e^	45.83 ± 0.67 ^h^	1.85 ± 0.03 ^h^	89.35 ± 0.20 ^e^	0.99 ± 0.09 ^ac^	7.69 ± 0.32 ^bd^	0.49 ± 0.00 ^a^
RJ3	49.01 ± 0.00 ^b^	74.39 ± 6.60 ^ab^	27.21 ± 0.06 ^a^	50.05 ± 0.00 ^abc^	4.04 ±0.06 ^a^	0.15 ± 0.00 ^bc^	0.28 ± 0.00 ^bc^	46.43 ± 1.63 ^e^	1.87 ± 0.04 ^e^	95.93 ± 0.55 ^b^	1.20 ± 0.26 ^abd^	8.53 ± 0.29 ^abc^	0.40 ± 0.01 ^i^
RB1	48.64 ± 0.02 ^d^	76.04 ± 6.35 ^b^	12.64 ± 0.15 ^a^	66.78 ± 0.00 ^d^	4.68 ± 0.17 ^a^	0.13 ± 0.00 ^e^	0.24 ± 0.01 ^bc^	45.81 ± 1.17 ^cd^	1.86 ± 0.06 ^cd^	87.85 ± 0.94 ^be^	1.36 ± 0.34 ^def^	8.35 ± 0.51 ^abcd^	0.37 ± 0.00 ^c^
RB2	49.31 ± 0.00 ^f^	87.85 ± 4.71 ^ac^	31.70 ± 0.08 ^a^	76.98 ± 0.00 ^ae^	7.65 ± 0.10 ^a^	0.11 ± 0.00 ^d^	0.21 ± 0.00 ^e^	47.62 ± 1.05 ^bc^	1.91 ± 0.03 ^bc^	88.65 ± 0.18 ^d^	1.07 ± 0.02 ^abc^	7.50 ± 0.36 ^d^	0.45 ± 0.01 ^f^
RB3	49.04 ± 0.01 ^g^	77.08 ± 3.21 ^abc^	22.60 ± 0.06 ^a^	40.40 ± 0.00 ^e^	3.72 ± 0.05 ^a^	0.16 ± 0.00 ^g^	0.30 ± 0.00 ^b^	46.67 ± 2.54 ^g^	1.88 ± 0.03 ^g^	91.32 ± 0.87 ^a^	1.09 ± 0.08 ^abc^	7.63 ± 0.31 ^d^	0.35 ± 0.00 ^e^
SJ1	49.17 ± 0.02 ^c^	79.49 ± 7.25 ^ab^	8.53 ± 0.10 ^a^	50.91 ± 0.01 ^ae^	3.95 ± 0.06 ^a^	0.16 ± 0.01 ^c^	0.30 ± 0.01 ^cd^	46.72 ± 2.95 ^e^	1.89 ± 0.12 ^e^	91.70 ± 0.45 ^a^	1.20 ± 0.08 ^abd^	7.93 ± 0.72 ^ac^	0.41 ± 0.00 ^g^
SJ2	49.27 ± 0.00 ^c^	85.11 ± 6.92 ^c^	33.68 ± 0.13 ^a^	74.97 ± 0.01 ^ab^	7.46 ± 0.12 ^a^	0.11 ± 0.00 ^a^	0.20 ± 0.00 ^a^	44.28 ± 1.05 ^a^	1.82 ± 0.03 ^a^	89.57 ± 0.38 ^a^	1.56 ± 0.13 ^f^	9.91 ± 0.58 ^e^	0.44 ± 0.00 ^c^
SJ3	49.29 ± 0.01 ^a^	74.54 ± 4.28 ^ab^	14.26 ± 0.15 ^a^	61.00 ± 0.01 ^cd^	4.20 ± 0.06 ^a^	0.14 ± 0.00 ^a^	0.26 ± 0.00 ^a^	46.17 ± 1.99 ^a^	1.88 ± 0.02 ^a^	94.91 ± 0.84 ^bc^	0.99 ± 0.09 ^ac^	8.57 ± 1.03 ^abc^	0.38 ± 0.01 ^h^
SB1	49.18 ± 0.02 ^h^	65.64 ± 6.45 ^ab^	18.11 ± 0.06 ^a^	54.55 ± 0.01 ^abe^	4.13 ± 0.06 ^a^	0.14 ± 0.01 ^a^	0.26 ± 0.00 ^a^	46.15 ± 0.93 ^a^	1.86 ± 0.03 ^a^	92.24 ± 0.96 ^ac^	1.19 ± 0.08 ^abd^	8.91 ± 0.62 ^a^	0.45 ± 0.00 ^d^
SB2	49.13 ± 0.02 ^a^	82.42 ± 7.30 ^ac^	38.38 ± 0.06 ^a^	63.93 ± 0.00 ^abc^	4.27 ± 0.11 ^a^	0.14 ± 0.00 ^f^	0.25 ± 0.01 ^a^	48.01 ± 1.05 ^f^	1.92 ± 0.03 ^f^	91.46 ± 0.19 ^d^	1.29 ± 0.06 ^bde^	8.57 ± 0.05 ^abc^	0.47 ± 0.00 ^b^
SB3	48.91 ± 0.01 ^b^	80.00 ± 4.52 ^ab^	13.51 ± 0.10 ^a^	73.00 ± 0.03 ^cd^	5.02 ± 0.06 ^a^	0.12 ± 0.00 ^d^	0.23 ± 0.00 ^d^	47.82 ± 3.66 ^b^	1.92 ± 0.09 ^b^	95.03 ± 0.49 ^d^	0.90 ± 0.07 ^c^	7.95 ± 0.35 ^bcd^	0.37 ± 0.01 ^ab^
S.E.M	0.00	54.0	0.000014	0.000037	3.44	0.0030	79.2	780.02	135.926	0.7	0.02080	0.281	0.000033
Oil	**	NS	**	**	NS	**	**	**	**	**	NS	**	**
MR	**	*	NS	NS	NS	**	**	**	**	**	**	*	NS
EO	**	NS	*	NS	NS	**	**	**	**	**	NS	**	**
Oil*MR	**	NS	NS	NS	NS	**	**	NS	NS	NS	**	**	**
Oil*EO	**	NS	**	NS	NS	**	**	*	NS	**	NS	NS	**
MR*EO	**	*	NS	NS	NS	**	*	**	**	**	NS	NS	**
Oil*MR*EO	**	NS	NS	NS	NS	**	*	**	**	NS	NS	NS	**

Results in this table are expressed as mean ± standard deviation. Mean values with the same superscript letters within a row are not significantly different at *p*  ≤  0.05. S.E.M.—standard error of the mean. *=p≤0.05; **=p≤0.001; NS—non−significant effect=p>0.05.

### 2.5. Particle Size Distribution

The PDI values ranged from 0.35 to 0.49 across all samples, with a trend of higher values observed in samples with an FB/CHP ratio of 2:1 (Table 1). This suggests that the faba bean protein content had a notable influence on particle size distribution. This observation aligns with the similarity between the PDI values of the microcapsules and those reported for pure broad bean protein (0.30–0.46), indicating the dominant role of this component in determining particle size uniformity [32]. All parameters and interactions between them (except MR) had a statistically significant impact on PDI values (*p* ≤ 0.001). Notably, the PDI values fell within the range indicative of relatively uniform particle size distribution, reflecting consistent sizing across the microcapsule samples [33,34]. The relatively uniform particle size distribution indicates the effectiveness of microencapsulation via a complex coacervation process in producing consistent-sized microcapsules, which is crucial for applications requiring standardized product characteristics.

### 2.6. Thermal Stability

The DSC analysis aimed to investigate the thermal behavior of the obtained microcapsules in the temperature range from 20 °C to 230 °C. The obtained results are presented in Table 2.

**Table 2:** Differential scanning calorimetry analysis results.

Sample	T_on_ [°C]	T_max_ [°C]	T_end_ [°C]	∆H [mJ]
**RJ1**	122.81 ± 0.01	133.79 ± 0.01	156.11	−523.77 ± 0.01
**RJ2**	154.70 ± 0.01	173.79 ± 0.01	195.12 ± 0.01	−438.73 ± 0.01
**RJ3**	87.01 ± 0.01	132.08 ± 0.01	157.47 ± 0.02	−361.65 ± 0.01
**RB1**	156.01 ± 0.01	158.99 ± 0.02	170.42 ± 0.02	−120.11 ± 0.00
**RB2**	177.52 ± 0.01	177.55 ± 0.02	181.40 ± 0.00	−428.38 ± 0.00
**RB3**	120.01 ± 0.02	135.35 ± 0.00	151.83 ± 0.01	−376.06 ± 0.01
**SJ1**	105.23 ± 0.00	133.89 ± 0.00	172.51 ± 0.01	−398.25 ± 0.00
**SJ2**	145.19 ± 0.00	151.69 ± 0.01	163.82 ± 0.01	−413.72 ± 0.00
**SJ3**	98.06 ± 0.01	134.72 ± 0.01	159.73 ± 0.01	−369.17 ± 0.01
**SB1**	128.33 ± 0.01	128.58 ± 0.01	174.08 ± 0.01	−466.23 ± 0.01
**SB2**	155.01 ± 0.01	165.76 ± 0.00	177.50 ± 0.01	−490.12 ± 0.01
**SB3**	56.80 ± 0.01	116.90 ± 0.02	155.52 ± 0.01	−392.99 ± 0.00
**FB**	48.97 ± 0.01	63.60 ± 0.01	76.94 ± 0.02	−37.27 ± 0.01
**CHP**	129.52 ± 0.02	139.62 ± 0.02	154.18 ± 0.01	−9663.97 ± 0.02
**JEO**	24.71 ± 0.00	89.17 ± 0.00	140.95 ± 0.01	−80.00 ± 0.01
**BPO**	26.81 ± 0.01	97.10 ± 0.02	148.11 ± 0.00	−92.36 ± 0.01
**RSO**	184.61 ± 0.01	199.29 ± 0.02	214.88 ± 0.02	102.75 ± 0.01
**SBO**	155.34 ± 0.01	174.31 ± 0.00	198.20 ± 0.02	108.87 ± 0.00
**T80**	116.40 ± 0.00	138.82 ± 0.02	154.42 ± 0.00	58.61 ± 0.00

First, the thermal behavior of all substances used to produce the microcapsules was analyzed individually. Broad bean protein exhibited an endothermic transition likely due to denaturation, commencing at 48.97 °C. This reaction peaked at T_max_ = 63.60 °C and concluded at 76.94 °C (∆H = −37.27 mJ). In a study by Buhler et al. [35], this reaction began at a significantly higher temperature, reaching its peak at 93.2 °C. The researchers noted that the initiation temperature of the reaction decreased with higher temperatures during the protein’s prior heating, attributed to partial denaturation of the protein during this process. Such substantial variations in observations may be attributed to the use of a pre-made preparation of broad bean protein in this experiment, which mainly contained soluble fractions. This factor could directly influence the reduction in the starting temperature of the protein denaturation reaction.

Polysaccharides from chia seeds displayed a single endothermic reaction commencing at T_on_ = 129.52 °C, corresponding to the loss of water and the decomposition of gums present in the mucus. This finding aligns with results reported by other researchers [36,37].

The endothermic reaction for juniper essential oil commenced at a temperature of T_on_ = 24.17°C, with a peak at T_max_ = 89.17 °C, and concluded at T_end_ = 140.95 °C, with an enthalpy change of ∆H = −80.00 mJ. Similarly, for black pepper essential oil, the endothermic peak was observed at T_on_ = 26.81°C, and the endothermic reaction reached T_max_ = 97.10 °C and concluded at T_end_ = 148.11 °C, with an enthalpy change of ∆H = −92.36 mJ. In both cases, this reaction can be attributed to the decomposition of EOs [38].

Both oils exhibited a single exothermic transformation, with RSO having a T_on_ of 184.61 °C and SBO having a T_on_ of 155.34 °C, corresponding to the formation of peroxides [39].

The emulsifier (T80) underwent a characteristic reaction with a flash point, characterized by T_on_ = 116.40 °C, T_max_ = 138.82 °C, T_end_ = 154.42 °C, and ∆H = 58.61 mJ [40,41].

Analysis of the obtained results reveals a clear influence of the mixing ratio on the thermal resistance of the produced microcapsules. Across all variants, it is evident that the initiation temperature of the reaction was lowest when FP/CHP = 2:1 and highest when FP/CHP = 1:2. Among all samples, RB2 demonstrated the highest thermal stability (T_on_ = 177.52 °C, T_max_ = 177.55 °C, T_end_ = 181.40 °C, ∆H = −428.38 mJ), whereas SB3 exhibited the lowest thermal stability (T_on_ = 56.80 °C, T_max_ = 116.90 °C, T_end_ = 155.52 °C, ∆H = −392.99 mJ). Notably, the initiation temperature of the reaction surpassed that of pure broad bean protein in all cases, while remaining below that of pure CHP for all samples. This underscores the interactions between the microcapsules’ wall materials, a phenomenon further supported by FT-IR analysis (Section 2.7.).

In summary, the results suggest that the optimal mixing ratio of faba bean protein and chia seed polysaccharides affects the thermal resistance of microcapsules, which is important for their potential applications in the food industry.

### 2.7. Fourier Transform Infrared Spectroscopy

**Figure 2:**
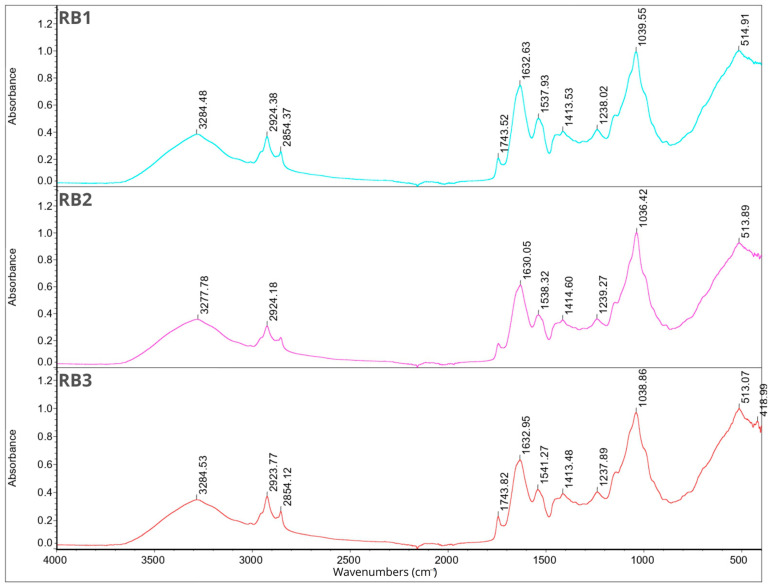
FT-IR spectra for samples RB1, RB2, RB3.

**Figure 3:**
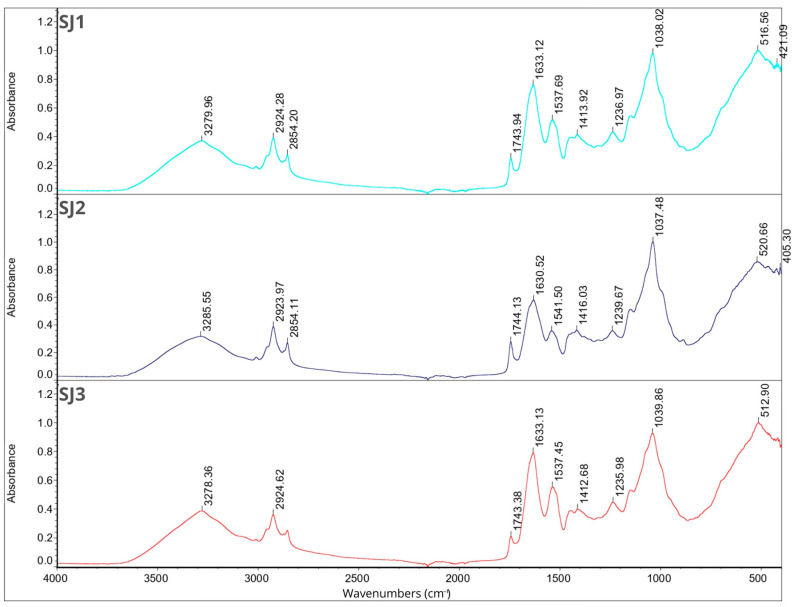
FT-IR spectra for samples SJ1, SJ2, SJ3.

**Figure 4:**
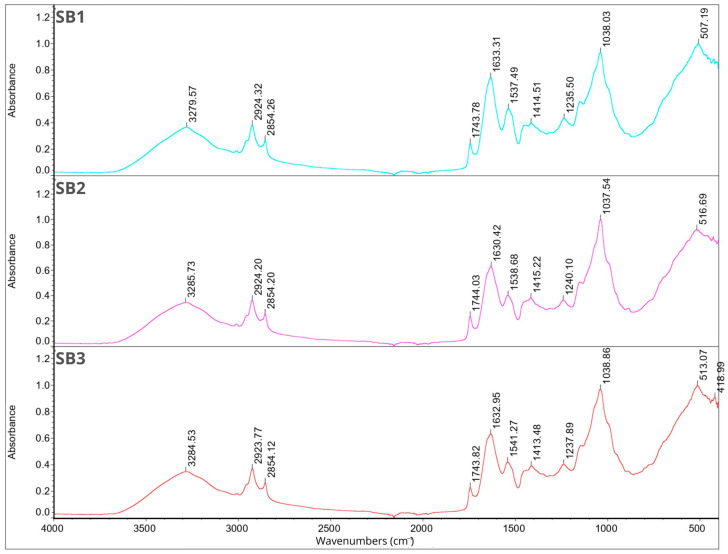
FT-IR spectra for samples SB1, SB2, SB3.

FT-IR analysis was performed for individual components of the microcapsules separately and for all 12 samples. The results are shown in Figure 1, Figure 2, Figure 3 and Figure 4. In the description of the results, the following markings were used for individual wavelength ranges: the single-bond area means waves with a length in the range of 4000–2500 cm^−1^, the triple-bond area means waves with a length in the range 2500–2000 cm^−1^, the double-bond area means waves with a length in the range 2000–1500 cm^−1^, and the fingerprint area means waves with a length in the range of 1500–600 cm^−1^ [42].

Nine characteristic peaks were identified for FB, confirming its complex nature (data not presented). In the region of single bonds, a broad absorption band at 3278.50 cm^−1^ suggests the presence of hydrogen bonds, indicating the existence of symmetric -OH stretching, ammonium, and amino groups. A peak at 2926.67 cm^−1^ is characteristic of the asymmetric stretching of C-H bonds in methylene groups [42]. This is a common feature in the FT-IR spectrum of proteins as methylene groups are found in amino acids such as alanine, leucine, isoleucine, valine, threonine, and methionine [43], which are components of broad bean protein [44]. A characteristic peak at 1633.37 cm^−1^, belonging to the amide I band (1700–1600 cm^−1^) [45], appeared, indicating stretching vibrations of C=C and C=O bonds. Additionally, in the amide II band (1510–1580 cm^−1^) [45], a peak was observed at 1536.95 cm^−1^, indicating bending in the N-H plane and stretching vibrations of C-N and C-C bonds. Within the fingerprint region, five peaks of varying intensities were identified: 1394.02 cm^−1^, 1236.99 cm^−1^, and 1043.14 cm^−1^, corresponding to bend vibrations of N-H and -NH_2_, and 513.95 cm^−1^ and 410.01 cm^−1^, corresponding to stretching vibrations of S-S derived from sulfur amino acids involved in disulfide bridge formation [46,47,48]. These findings are consistent with previous studies conducted by Sofi et al. [22].

Fourteen peaks were identified in the FT-IR spectrum of CHP (data not presented). A broad absorption band at 3472.39 cm^−1^ indicates the presence of hydrogen bonds, suggesting symmetric -OH stretching and thus indicating the water content in the sample [31,36]. Peaks at 2927.61 cm^−1^ and 2355.77 cm^−1^ indicate C-H stretching. Peaks at 1629.67 cm^−1^ and 1537.45 cm^−1^ belong to amide I, corresponding to C=C and C=O bonds. Peaks at 1449.60 cm^−1^ and 1399.72 cm^−1^ are attributed to symmetrical -COO-link vibrations associated with uronic acids present in CHP [49]. Peaks at 1237.04 cm^−1^ and 1156.27 cm^−1^ indicate O-C-O asymmetric stretching, potentially associated with xyloglucan presence [36,50]. Peaks at 1063.49 cm^−1^ and 1029.91 cm^−1^ are related to the C-O bond, bend vibrations of N-H and -NH_2_, and large aromatic rings [42]. Subsequent peaks at 856.07 cm^−1^ and 801.47 cm^−1^ correspond to anomeric configurations, such as CH oscillations of α and β conformers and glycosidic linkages, attributed to α-D-galactopyranose units and β-D-mannopyranose units, respectively [36,50]. The last peak, identified at 661.10 cm^−1^, may be attributed to CH_2_ bonds originating from chains containing more than seven carbon atoms [49].

The FT-IR spectra of JEO exhibit distinctive peaks indicative of its chemical composition (data not presented). A prominent peak at 3387.94 cm^−1^ signifies the presence of phenolic -OH groups. Peaks at 2917.56 cm^−1^ (the most intense peak), 2878.29 cm^−1^, and 2833.70 cm^−1^ suggest the presence of long-chain linear aliphatic compounds (C-H stretching) [42]. The peak at 1748.55 cm^−1^ corresponds to C=O bonds, while the peak at 1644.81 cm^−1^ corresponds to C=C bonds. Peaks at 1594.93 cm^−1^ and 1515.09 cm^−1^ are associated with C=C bonds from aromatic rings [42,51]. The fingerprint region shows peaks of varying intensity, including those at 1446.07 cm^−1^, 1364.69 cm^−1^, and 1328.55 cm^−1^, corresponding to C-OH bending vibrations and methyl C-H symmetrical and asymmetrical bends [51,52]. The peak at 1264.26 cm^−1^ is attributed to C-C-O and C-O stretching vibrations of phenolics, while peaks at 1164.67 cm^−1^, 1124.97 cm^−1^, and 1101.48 cm^−1^ result from C-O and C-OH bond deformation vibrations [51,52,53]. Peaks at 1063.07 cm^−1^ and 1014.59 cm^−1^ can be assigned to hydroxyl group vibrations and methylene vibrations, while peaks at 989.83 cm^−1^ and 952.06 cm^−1^ represent C-H bending vibration absorptions. Peaks at 887.14 cm^−1^ (the third most intense), 814.45 cm^−1^, 786.52 cm^−1^ (the second most intense), 618.60 cm^−1^, and 418.96 cm^−1^ suggest the presence of aromatic rings [3,42].

For BPO, peaks in the single-bond area at 2954.86 cm^−1^, 2922.95 cm^−1^ (the most intense peak), and 2867.06 cm^−1^ indicate C-H stretching in long-chain linear aliphatic compounds. The peak at 1643.05 cm^−1^ may relate to H-O-H bending, while another peak at 1446.24 cm^−1^ corresponds to C-OH bending [54]. Peaks at 1381.29 cm^−1^ and 1366.31 cm^−1^ are associated with symmetrical deformation of CH_3_ [42,55]. The peak at 1276.14 cm^−1^ represents skeletal C-C vibrations. Peaks at 1181.19 cm^−1^ and 1023.33 cm^−1^ may be attributed to stretching vibrations of terpenoid components, while 986.94 cm^−1^ may be related to the asymmetric stretching of the C-O bond [54]. Peaks at 885.83 cm^−1^ (the second most intense) and 875.20 cm^−1^ (the third most intense) suggest the presence of C-H stretching vibrations of aromatics [56]. The peak at 786.32 cm^−1^ is associated with S-C absorption. Finally, peaks at 543.65 cm^−1^, 421.49 cm^−1^, and 442.11 cm^−1^ suggest the presence of aromatic rings [3,42] (data not presented).

Triacylglycerols are the primary constituents of oils; hence, they dominate the spectrum in the FT-IR analysis of rapeseed and soybean oil. Peaks identified for RSO include 3076.53 cm^−1^ corresponding to the C-H stretching of cis double bonds, 2923.53 cm^−1^ and 2853.29 cm^−1^ corresponding to the methylene C-H asymmetrical stretching, 1743.07 cm^−1^ corresponding to the ester C=O stretching, a prominent peak at 1629.91 cm^−1^ assigned to the cis C=C stretching, 1530.48 cm^−1^, 1454.27 cm^−1^, and 1401.01 cm^−1^ corresponding to the H-C-H and =C-H (-cis) bending, and peaks at 1235.43 cm^−1^, 1156.58 cm^−1^, and 1045.80 cm^−1^ assigned to the ester C-O stretching. These findings are in line with previous research findings [42,57]. For SBO, peaks at similar wavelengths were observed, corresponding to the same bonds as in the case of RSO: 3008.58 cm^−1^, 2922.40 cm^−1^, 2852.54 cm^−1^, and 1742.92 cm^−1^. Additionally, peaks at 1456.95 cm^−1^ and 1377.02 cm^−1^ are attributed to the -CH_2_ bending of cis C=C bonds, while peaks at 1159.01 cm^−1^ and 1097.76 cm^−1^ are attributed to C–O stretching vibrations. Furthermore, a peak at 720.99 cm^−1^, attributed to the -CH_2_ rocking, was identified [42,58].

The analysis of the Tween 80 emulsifier revealed an initial peak at 2855.75 cm^−1^ that may signify C-H bending. Peaks in the 1470–720 cm^−1^ range suggest alignment with long-chain linear aliphatic compounds and -OH bending. The subsequent peak at 1734.71 cm^−1^ may correspond to aromatic bands. No indications of triple bonds were observed. Peaks in the fingerprint region (1456.94 cm^−1^, 1348.54 cm^−1^, 1296.36 cm^−1^, 1247.46 cm^−1^, 1096.21 cm^−1^, 946.67 cm^−1^, 846.64 cm^−1^, and 510.58 cm^−1^) confirmed the presence of aromatic rings and double bonds (data not presented). The obtained results are consistent with previous studies [41,42,59].

Figure 1, Figure 2, Figure 3 and Figure 4 illustrate the FT-IR spectra for all obtained microcapsules. The absence of characteristic peaks for RSO, SBO, JEO, and BPO in the spectra suggests successful microencapsulation, corroborated by EE test results (Section 2.1., Table 1). The spectra closely resemble those of FB, consistent with their composition, with the FB concentration being the highest in all cases. Variations in peak intensity were observed, notably with higher intensity at specific wavelengths for certain mixing ratios. Notably, a peak characteristic of RSO and SBO appeared in all samples except RB2, which exhibited the highest EE (87.85 ± 4.71). This indicates a correlation between microcapsule composition, peak intensity, and encapsulation efficiency.

### 2.8. Smell Pattern

**Figure 5:**
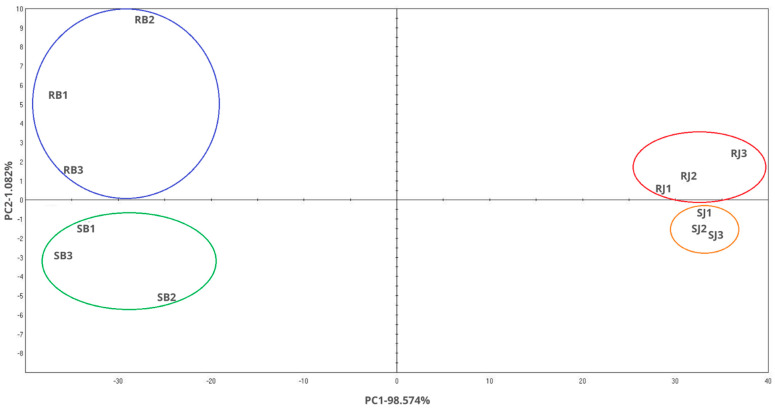
Smell pattern chart.

Figure 5 illustrates the classification of scent profiles relative to their respective experimental groups. The samples are depicted in a two-dimensional plane based on principal component 1 (PC1) and principal component 2 (PC2). The total contribution variances of PC1 and PC2 from direct electronic nose measurements were 98.574% and 1.082%, respectively. The differentiation index (DI) was 100%, indicating clear discrimination among the samples. Notably, the samples were well separated with no overlapping areas, underscoring significant differences among them. Interestingly, samples containing black pepper essential oil were predominantly positioned on the left side of the chart, while those with juniper essential oil were primarily located on the right side. Additionally, samples containing RSO tended to cluster in the upper part of the graph, whereas those with SBO were mainly situated in the lower part. Each group exhibited distinct separation from one another, as indicated by the color markings on the chart. The distinct separation of scent profiles based on experimental groups suggests that the electronic nose technique effectively discriminates between different sample compositions. The observed clustering patterns could be attributed to the unique chemical compositions of the essential oils and carrier oils used in each sample, highlighting the potential influence of these components on scent profiles.

## 3. Materials and Methods

### 3.1. Materials

Faba bean protein (Hortimex, Konin, Poland) and polysaccharides extracted from chia seeds (Agnex, Białystok, Poland) were employed as wall materials in this study. Juniper berry essential oil (*Juniperus communis*) and black peppercorn essential oil (*Piper nigrum*) (Ancient Wisdom, Sheffield, United Kingdom) were initially dissolved in soybean oil (Dary Natury, Koryciny, Poland) or rapeseed oil (Olvita, Marcinowice, Poland) and utilized as core materials. Samples were prepared with the addition of the emulsifier Tween 80 (Sigma Aldrich, Saint Louis, MO, USA).

### 3.2. Chia Polysaccharide Extraction

Chia seeds were combined with distilled water at room temperature, using a ratio of 5 g of seeds per 100 g of water. Polysaccharide extraction took place at room temperature over a 4 h period, with constant stirring. Following extraction, the polysaccharides were filtered through cheesecloth, subjected to centrifugation at 10,000 rpm for 5 min, and then freeze-dried for 72 h. The resulting mucilage, prepared using a slightly modified version of the methodology outlined by Silva et al. [31], was promptly employed for the subsequent coacervation process (Section 3.3.).

### 3.3. Preparation of Coacervates

A 5% solution of faba bean protein (FB) was mixed with a 2% solution of chia seed polysaccharides (CHP) (*w*/*v* in double distilled water) in various mass ratios (1:1; 1:2, and 2:1) to obtain 300 g of solution. The mixture of wall materials prepared in this way with 0.5% Tween 80 was mixed on a magnetic mixer for 10 min until the solutions were completely combined with the emulsifier. After this time, a mixture of soybean (SBO) or rapeseed (RPO) oil with juniper (JEO) or black pepper (BPO) essential oil was added in an amount of 5% of the total sample weight. Each sample contained 2.5% oil and 2.5% essential oil. After initial mixing on a magnetic stirrer, the samples were subjected to high shear homogenization using an Ultra Turrax (IKA T18 basic, IKA, Staufen, Germany) for 10 min at 15,000 rpm/min at room temperature. After emulsification, the pH was adjusted to 4.0 (below the isoelectric point) using 1 M HCl. Subsequently, all emulsions were stored at 4 °C for 24 h, followed by transfer to −20 °C for an additional 24 h, and then transferred again to −60 °C for another 24 h. The frozen samples were then subjected to lyophilization for 72 h at −80 °C. Following lyophilization, the lyophilizates were sieved using a laboratory sieve with a mesh size of 710 μm, vacuum packed, and stored at 4 °C for further analyses (Table 3).

### 3.4. Microcapsule Yield and Encapsulation Efficiency

Using the mass of liquid (LC) and dried (DC) coacervates, we calculated the actual yield of microcapsules (MY). All measurements were performed in triplicates.

$$MY=\frac{DC}{LC}*100\%$$

To determine the encapsulation efficiency (EE), we used the methodology from our previous study [60]. Briefly, the encapsulation efficiency was determined as the ratio of internal oil to the total oil content of the samples. Total oil (TO) and surface oil (SO) were measured in triplicate for each sample.

$$EE=\frac{TO-SO}{TO}*100\%$$

To calculate SO, the sample was dissolved in 30 mL of n-hexane, filtered, and evaporated (R-100, Büchi, Flawil, Switzerland). The remaining oil mass after evaporation was subtracted from the theoretical weight of oil from the sample. For determining TO, the sample (1.5 g) was mixed (60 rpm, 15 min) with 4 mL of KCl, 8 mL of acetone, and 8 mL of chloroform. After centrifugation (10,000 rpm, 10 min), the chloroform layer containing the extracted oil was filtered and evaporated. The remaining oil mass after evaporation was then measured.

### 3.5. Density, Carr Index, and Hausner Ratio

Bulk density (ρ_bulk_) was assessed by pouring 1 g of the sample into a 10 mL graduated cylinder and measuring the volume it occupied. Tapped density (ρ_tap_) was determined by manually tapping the cylinder repeatedly for one minute at a vertical distance of 14 ± 2 mm. Both measurements were conducted in triplicate, and densities were expressed in g/cm^3^. The Compressibility Index (CI) and Hausner ratio (HR) were calculated from the obtained results to evaluate the powder’s flowability and compressibility [28].

$$CI=\frac{\rho_{tap}-\rho_{bulk}}{\rho_{tap}}*100\%$$

$$HR=\frac{\rho_{tap}}{\rho_{bulk}}$$

### 3.6. Solubility, Moisture Content, and Hygroscopicity

The solubility of the powders was evaluated using a method involving the dispersion of 0.5 g of the sample in 50 mL of double-distilled water. The mixture was then agitated for 30 min at 60 rpm before undergoing centrifugation at 10,000 rpm for 5 min. After centrifugation, 25 mL of the supernatant was carefully transferred onto a pre-weighed Petri dish and dried at 105 °C for 6 h using a Binder FP 115 drying oven from Tuttlingen, Germany. The solubility (%) was calculated as the percentage of dried supernatant relative to the initially added amount of powder, following the procedure outlined by De Melo Ramos et al. [61]. All measurements were performed in triplicate to ensure the accuracy and consistency of results.

The moisture content of the obtained powders was determined by placing 0.2 g of the sample in a pre-weighed Petri dish. The dish with the sample was then dried at 70 °C for 24 h (Binder FP 115 drying oven, Tuttlingen, Germany). Subsequently, the dried samples were transferred to a desiccator to cool completely before being re-weighed. The moisture content was calculated based on the observed difference in weight before and after the drying process (%) [62]. Determinations were performed in triplicate.

The hygroscopicity of the obtained powders was determined by placing 0.2 g of the sample in a pre-weighed Petri dish. The dish was then stored in a desiccator containing a saturated Na_2_SO_4_ solution for a duration of one week. Hygroscopicity was expressed as g of water absorbed per 100 g sample (%) [62]. Determinations were performed in triplicate.

### 3.7. Color Measurement

The color of the microcapsules was assessed using a Minolta CR-400 colorimeter manufactured by Konica Minolta Inc., Tokio, Japan. The measurements were conducted under D65 illuminant conditions, with an 8 mm measuring surface and following the standard 2° observer protocol. The recorded data were expressed in accordance with the International Commission on Lighting’s (Commission Internationale de L’Eclarige) system within the CIELab color space.

The parameters analyzed and evaluated included L* (where L = 0 represents black and L = 100 represents white), a* (where −a signifies green and +a signifies red), and b* (where −b represents blue and +b represents yellow) [63]. These determinations were performed in triplicate immediately after the production process to ensure accuracy and consistency.

### 3.8. Particle Size Distribution

The particle size analysis was carried out using the Morphologi^®^ G3SE instrument from Malvern Instruments Ltd., headquartered in Malvern, UK. The instrument was outfitted with a dispersion unit tailored for dry samples. Particle size distribution was determined by assessing the relative volume of particles within predefined size ranges, as illustrated in the size distribution curves. Data analysis was conducted using Malvern Microsoftware v.5.40, proprietary software provided by Malvern Instruments Ltd. Additionally, the particle size distribution (span index—SI) was estimated using the formula outlined by Hernandez-Nava et al. [64]:

$$SI=\frac{D_{90}-D_{10}}{D_{50}}$$

where D_90_, D_50_, and D_10_ are the equivalent volume diameters at 90%, 50%, and 10% cumulative volume, respectively.

### 3.9. Thermal Stability

The thermal properties of the samples were evaluated using a differential scanning calorimetry instrument (DSC 1) from Mettler Toledo 820 (Schwerzenbach, Switzerland) under a nitrogen atmosphere at a flow rate of 100 cm^3^/min, as per the method described [21] with some modifications. The instrument was calibrated with pure indium and zinc. Each sample (5.0 ± 0.1 mg) was placed in an aluminum crucible (ME-51119870) and covered with a lid (ME-51119871) using the Mettler Toledo Crucible Sealing Press. DSC scans were recorded from 10 °C to 230 °C at a rate of 10 °C/min. The thermograms were analyzed using STARe Software (Version 9.30) to determine the start (T_on_), maximum (T_max_), and end (T_end_) temperatures, as well as the areas under the peaks (ΔH).

### 3.10. Fourier Transform Infrared Spectroscopy (FT-IR)

FT-IR spectra were recorded on a Nicolet™ iS™ 5 FTIR Spectrometer (Thermo Scientific, Waltham, MA, USA), with a horizontal device for attenuated reflectance and diamond crystal, on a spectral window ranging from 4000 to 400 cm^−1^, at a spectral resolution of 2 cm^−1^. Spectra were recorded without any sample preparation and were processed with the OMNIC program (Thermo Scientific, Waltham, MA, USA).

### 3.11. Smell Pattern

The volatile compounds within the microcapsules were extracted using the Heracles II electronic nose (Alpha M.O.S., Toulouse, France), which utilizes ultra-fast gas chromatography with headspace. The system features a detection system comprising two metal columns of varying polarities (nonpolar MXT-5 and slightly polar MXT1701, diameter = 180 µm, length = 10 m) and two flame ionization detectors (FIDs).

For the analysis, 10% solutions (0.25 g in 5 g) of each sample were placed in standard headspace vials sealed with a Teflon-faced silicon rubber cap. Incubation was performed at 35 °C for 900 s under an agitation speed of 8.33 Hz. The carrying gas was hydrogen (flow rate 1 mL/min). The injector temperature was set at 200 °C, with an injected volume of 3500 µL and speed of 125 mL s^−1^. The analytes were collected in the trap at 15 °C and subsequently divided and simultaneously transferred into the two columns. The carrying gas was maintained at a constant pressure of 80 kPa, with a split flow rate of 10 mL/min at the column heads. The temperature program in the oven was as follows: 60 °C for 2 s, a ramp of 3 °C s^−1^ to 270 °C, held for 20 s, and FID1/FID2 at 280 °C.

The volatile compounds identified in the samples were presented in the form of a table with Kovats indices. All samples were analyzed in triplicate. Kovats indices were established using alkane standards (n-butane to n-hexadecane) (Restek Centre County, PA) measured under the same conditions as the samples [65,66].

### 3.12. Statistical Analysis

For statistical analysis, STATISTICA (v. 13.3) software was used. To check the impact of the mixing ratio, oil, and essential oil content on obtained results and significant differences between them, factorial ANOVA and Fisher LSD test (*p*-value < 0.05, α = 95%) were used.

## 4. Conclusions

In this study, microcapsules containing essential oils were developed using broad bean protein and chia seed polysaccharides at various ratios (1:1, 1:2, or 2:1). The results revealed that the encapsulation efficiency of the microcapsules ranged from 65.64% to 87.85%, indicating their potential for industrial applications. Particularly noteworthy was the highest encapsulation efficiency observed in samples with a protein-to-polysaccharide ratio of 1:2. Another significant finding was the impact of water content on the density and hygroscopicity of the microcapsules, where an increase in water content resulted in decreased density and increased hygroscopicity. This observation was corroborated by FT-IR analysis. Additionally, the microcapsules exhibited varying degrees of temperature resistance, with the most thermally stable ones observed at a protein-to-polysaccharide ratio of 1:2. These findings underscore the importance of the ratio of broad bean protein to chia seed polysaccharides in determining encapsulation efficiency, with the 1:2 ratio being optimal. Moreover, this ratio facilitated the production of microcapsules with enhanced thermal stability, a crucial factor considering the presence of volatile compounds like essential oils. Lastly, the influence of water content on microcapsule properties emphasizes the necessity of considering this factor during the production process design.

## Figures and Tables

**Table 3:** Coding of samples.

Oil	Essential Oil	FP/CHP	Code
Rapeseed	Juniper	1:1	RJ1
		1:2	RJ2
		2:1	RJ3
	Black pepper	1:1	RB1
		1:2	RB2
		2:1	RB3
Soybean	Juniper	1:1	SJ1
		1:2	SJ2
		2:1	SJ3
	Black pepper	1:1	SB1
		1:2	SB2
		2:1	SB3

## Data Availability

The raw data supporting the conclusions of this article will be made available by the authors on request.
